# Medical Faculty Perspectives Toward Cadaveric Dissection as a Learning Tool for Anatomy Education: A Survey Study in India

**DOI:** 10.7759/cureus.37713

**Published:** 2023-04-17

**Authors:** Mohammad R Asad, Adil Asghar, Nasir Tadvi, Mohammad M Ahmed, Mohammed Nazeer, Khwaja M Amir, Nazim Nasir, Riyaz A Shaik, Apurba Patra

**Affiliations:** 1 Department of Basic Medical Sciences, College of Medicine, Majmaah University, Al Majmaah, SAU; 2 Anatomy, All India Institute of Medical Sciences, Patna, IND; 3 Department of Pharmacology, Government Medical College, Telengna, IND; 4 Department of Basic Medical Sciences, Majmaah University, Al Majmaah, SAU; 5 Department of Basic Medical Sciences, College of Applied Medical Sciences, King Khalid University, Abha, SAU; 6 Department of Family and Community Medicine, College of Medicine, Majmaah University, Al Majmaah, SAU; 7 Anatomy, All India Institute of Medical Sciences, Bathinda, IND

**Keywords:** virtual anatomy, empathy, learning styles, anatomy education, cadaveric dissection

## Abstract

Cadaveric dissection, as a learning tool, has been a part of Indian medical education. Worldwide, with reforms in medical education and the introduction of new learning modalities, cadaveric dissection has been complemented with other modalities such as living anatomy and virtual anatomy. This study aims to collect the feedback of faculty members regarding the role of dissection in the present context of medical education. The method of the study involved a 32-item questionnaire to collect responses; they were collected using the 5-point Likert scale along with two open-ended questions. In general, the closed questions covered these sections: learning styles, interpersonal skills, teaching and learning, dissection, and other learning modes. The principal component analysis was used to explore the multivariate relationships among the items’ perceptions. The multivariate regression analysis was conducted between the construct and the latent variable to develop the structural equation model. Four themes, PC1 (learning ability with structural orientation), PC2 (interpersonal skill), PC3 (multimedia-virtual tool), and PC5 (associated factors) had positive relation and were treated as a latent variable motivation for dissection, and theme 4 (PC4, safety) had a negative correlation and was treated as a latent variable repulsion for dissection. It was found that the dissection room is an important place for learning clinical and personal skills, along with empathy, in anatomy education. Safety issues and implementation of stress-coping activities during the induction phase are required. There is also a need to use mixed-method approaches that integrate technology-enhanced learning such as virtual anatomy, living anatomy, and radiological anatomy with cadaveric dissection.

## Introduction

Anatomy education is all about understanding the structure, its relations, and functions. Recently, there has been a paradigm shift; old and traditional cadaveric dissection has been superseded with virtual dissectors and three-dimensional models. Cadaveric dissection has become the primary learning tool for anatomy in medical education in the last four centuries [1]. Shifting medical education from traditional didactic learning to integrated curricula has reduced the allocated hours for anatomy teaching worldwide [2].

Indian context

The first modern medical school in India has its origin in Calcutta (1835). The first cadaveric dissection as part of anatomy education took place a year later, in 1836, in the same medical school [3]. Recently, the National Medical Commission (NMC) has implemented a competency-based undergraduate curriculum for Indian medical undergraduates (CBME). This is a major shift from the traditional method of teaching anatomy to ensure integration among different disciplines. Moreover, this newer curriculum is expected to focus on developing the necessary skills and attitudes required for a clinician to practice in the community. Four hundred and nine learning outcomes of anatomy have been divided into four learning domains (knowledge, skills, attitude, and communication) to ensure vertical integration with surgery, medicine, orthopedics, and radiology. Viva-voce/skill assessments are aligned as assessment modalities with practical sessions [4]. The Indian medical curriculum is subject-based and divided into the preclinical, paraclinical, and clinical phases. A uniform syllabus, as directed by NMC, is followed across the country. Amongst preclinical and paraclinical subjects, the maximum contact hours have been allocated to anatomy in the Indian context, i.e., 650 hours [5]. More than two-thirds of the allocated hours have been utilized by practical sessions where cadaveric dissection is one of the main learning modalities [6]. There is also an argument that the act of dissection facilitates empathy, professionalism, and a team-based approach among undergraduates at an early stage of their medical carrier, thereby addressing competencies of affective domains [7]. In developing countries, cadaveric dissection seems to play a pivotal role in teaching anatomy. In new system-based curricula, prosected and plastinated specimens’ adjunct with other approaches, such as body painting, imaging, and computer-assisted learning, has replaced dissection to a more considerable extent [ 8-9]. A combined approach of virtual dissector with other e-learning tools is taking space in anatomy education. Medical students need to be competent and confident in order to work with these newer technologies and curricula. On the other hand, Indian medical schools are also facing challenges in procuring dead bodies for dissection due to the lack of a structured body donation program, and some of them have curtailed dissection due to the unavailability of cadavers [10].

Modern vs. traditional approaches in anatomy learning

Modern teaching methods have substituted the traditional teaching of anatomy conducted by didactic lectures, tutorials, and cadaveric dissection such as prosection, problem-based tutorial, self-directed learning, computer-assisted learning, and plastinated models [11-12]. Dissection, which inculcates the basics of medicine among undergraduates, is gradually disappearing from the modern curriculum. Curricular reforms, high financial and infrastructural demand for dissection halls, physical and psychological health hazards associated with dissection, and ethical issues were also reported as the factors responsible for the decline in dissections [13]. Dissection enhances manual dexterity, communication skills, and the concept of biological and pathological variation [14]. It also helps to develop teamwork, peer-mediated and self-directed learning, three-dimensional appreciation of the human body with a problem-solving approach, and acclimatization to death and patient [15]. There is a debate over the role of dissection in anatomy education in medical colleges in recent times. The first view asserted that dissection is an excellent teaching of anatomy mode [15]. Such opinion is mainly held by surgeons, anatomists, and medical students [15-16]. The second point of view asserted that dissection could be removed from anatomy education [17]. This view has been supported mainly by an educationist who favors new teaching methods, such as problem-based learning, computer-assisted learning, and self-directed learning [15-16]. There has been a view that dissection of embalmed bodies leads to difficulty in some students due to short-term psychological stress and exposure to the irritant and carcinogen formalin. However, the studies comparing the competency of dissection versus newly introduced technological resources suggested retention of dissection complemented with recently introduced teaching methods [18].

Notwithstanding the arguments for and against using anatomical dissection as a teaching tool, pertinent impartial research has not been examined. Learning from human cadavers is a challenging learning experience with many elements that are difficult to measure and evaluate impartially [16]. The unavailability of appropriate data has been repeatedly misleading.

Most of the stakeholders’ survey studies regarding cadaveric dissection in the Indian context have included student perceptions; however, none of them has considered faculty perception. This survey includes a faculty member of anatomy. It also collects in-depth views of faculty members regarding the impact of cadaveric dissection as a teaching and learning tool, along with a suggestion for improvements from their comments by using open-ended questions.

The current engagements with virtual dissectors and 3D models show that it is difficult to interact with virtual dissectors like cadaveric dissection. The Indian medical learners are late recipients of these newer technologies, and they were only exposed to traditional cadaveric dissection. The transition between the two and the lack of proactive or devoted manpower caused a serious impact on learning and development for competence and confidence. The objective of the current study was to find out if there was a relationship between learning attributes and motivation for dissection based on faculty perceptions.

## Materials and methods

Participants

The study used a quantitative cross-sectional self-reporting questionnaire that collected demographics and measured the learning behavior associated with cadaveric dissection: ability, structural orientation, interpersonal skill, and the inclusion of multimedia-virtual tools. The outcomes were measured in the anatomy faculties of India working in different medical institutions. The responses were collected from the medical education Google group of Indian faculty members;  is an online platform having 4,000 Indian faculty members across the country for discussing issues and sharing ideas regarding Indian medical education. A separate Google account was created for data collection, only having access to the research project members. The link to the survey was emailed from the research account to the moderator of the meu_india group. In total, 556 responses were collected in two months from out of 4,000 faculty members in the group.

Inclusion criterion

Experience of teaching as faculty in India’s graduation medical program was considered the inclusion criterion for the participants. Any Indian medical faculty with a minimum of one year of teaching experience or more was eligible for filling out the questionnaire.

Ethical statement

Ethical approval was taken prior to the study. The approval number was AIHMS/VII/34. Informed consent was taken from each of the participants. The last section of the questionnaire was regarding the consent and the permission of the participants to use the data for medical education research.

Questionnaire

A 32-item questionnaire divided into five sections was used for the survey. The first section pertains to general details, including gender, role as a faculty member, and years of experience. The subsequent sections were divided into the following categories: learning styles, interpersonal skills, teaching and learning, dissection, and other learning modes. The 5-point Likert scale was used to collect the responses based on a scale from zero to four, ranging from "strongly disagree" to "strongly agree." During the construction of the questionnaire, extensive literature was searched using the keywords cadaveric dissection and anatomy teaching and learning modalities in anatomy by using MEDLINE and other search engines. The items were peer-reviewed by experts (medical educationists and anatomists) to ensure the questionnaire's content and face validity. The internal consistency and reliability of each section of the questionnaire were calculated separately: learning styles (0.694), interpersonal skills (0.82), teaching and learning (0.676), dissection, and other learning modalities (0.758) by the split-half method. Using Cronbach’s alpha and McDonald’s omega, the permutations of the questionnaire were tested for internal consistency or reliability. Some of the items may be coded in reverse for the negative correlation.

Statistical analysis

Excel and R-package were used for the quantitative and qualitative estimations. Each item was considered a construct or manifest variable, and the section in which the item belonged was a latent variable. Mean, median, SD, and IQR were calculated. A t-test or Mann-Whitney U test was applied for intergroup comparisons. The entire data were examined using the Shapiro-Wilk test for normal distribution. Again, the reliability index of permutation was measured by dropping each item. The data were split into two halves based on odd and even sequences. The odd sequences were utilized for exploratory analysis by principal components analysis. Subsequently, the principal component analysis, a data reduction technique, was used to explore the multivariate relationships among the items’ perceptions. It would reveal any correlation between the learning attributes and perception of dissection. The PCA was run on the data. The Kaiser-Meyer-Olkin test for sample adequacy and Bartlett’s test of sphericity for model fit or assumption check were applied. The multicollinearity of data was tested. The components were extracted using the criteria for eigenvalues (1.5) and examined the Scree plot. A predicted model was developed based on path analysis utilizing the principal components. Then, the predicted model was re-examined for model fit by confirmatory analysis. Then, the latent variable analysis of the principal components was conducted to finalize the predicted model as the structural equation model. The same would also calculate the covariance and residual parameters of the construct and latent variables.

Confirmatory factor analysis and model fit

The even sequences were used for confirmatory factor analysis. A latent path model was developed based on theoretical consideration and the result of the principal component analysis, analysis of variance, and correlation analysis. The model fit was tested by least square estimation. The comparative index, Tucker-Lewis index, root mean square error of approximation, and standardized root mean square residual were computed for model fit. Latent variable analysis was conducted to estimate the relationship between construct and latent variables with years of dissection experience. The multivariate regression analysis was performed between the construct and latent variables to develop the structural equation model.

## Results

**Figure 1:**
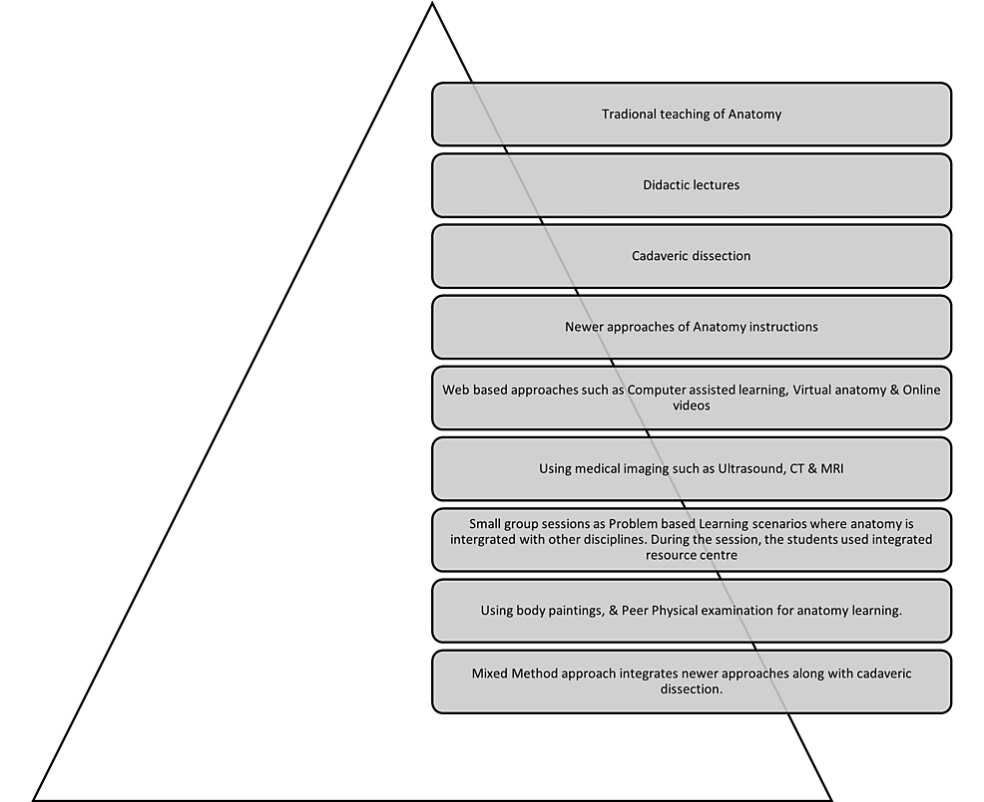
Instructional methods used in traditional vs. new approaches to anatomy learning along with mixed method approach

Figure 1 shows the instructional methods used in traditional vs. new approaches to anatomy learning, as well as mixed-method approaches.

Sample description

The sample size was 484, representing a completion rate of 87% (484/556). The descriptive statistics of the sample are presented in Table 1. The median age was 51 years, ranging from 28 to 70. No significant difference in perception was found in both genders in most items except a few, like empathy and integration of dissection with living anatomy (Table 1). Perception of empathy was observed to be significantly higher in female faculty members; male members had a significantly higher perception of integration of dissection with radiological, living, and surface anatomy. The perceptions were measured for different survey items using the ANOVA based on years of exposure to cadaveric dissection. The faculties with long exposure to dissection rated significantly higher for empathy and positive behavior development (Table 1). Most of the faculties that had prolonged exposure to dissection have rated considerably higher for virtual anatomy's cost-effectiveness.

**Table 1:** Descriptive statistics of participants and items Item codes (questionnaire sections): L1- L4 (learning styles), I1- I5 (interpersonal skills), TL1-TL7 (teaching and learning), DL1-DL8 (dissection as a learning tool)

Item code	Question	Total	Mean±SD		Dissection exposure (yrs)			
			Male	Female	0-2 yrs.	2-5 yrs.	5-10 yrs.	> 10 yrs.
	Mean age (yrs) range (28-70 yrs)	47.4±20.1	48.7±21.2	44.8±19.8	32.1±17.8	34.3±11.9	41.8±27.4	52.8±26.9
	Sample	(n=484)	258	226	86	102	110	186
L1	Cadaveric dissection is an effective teaching tool for visual learning	3.64±0.79	3.59±0.8	3.7±0.67	3.58±0.73	3.73±0.57	3.58±0.9	3.66±0.73
L2	Cadaveric dissection is an effective teaching tool for auditory learning	2.55±1.32	2.55±1.23	2.56±1.25	2.72±1.14	2.51±1.07	2.67±1.32	2.43±1.31
L3	Cadaveric dissection is an effective teaching tool for kinesthetic learning (learning by doing)	3.62±0.79	3.6±0.81	3.62±0.66	3.44±0.8	3.61±0.8	3.56±0.94	3.72±0.52
L4	Cadaveric dissection is an effective teaching tool for developing reading and writing skills	2.24±1.34	2.08±1.4	2.4±1.3	2.07±1.18	2.43±1.2	2.04±1.5	2.32±1.4
I1	Cadaveric dissection sessions facilitate the enhancement of leadership skills	2.64±1.29	2.64±1.24	2.66±1.19	2.47±1.26	2.65±1.21	2.71±1.32	2.69±1.13
I2	Cadaveric dissection sessions facilitate positive behavior modification toward the colleagues	2.88±1.14	2.8±1.08	2.97±1.07	2.44±1.12^$^	2.78±1.01^$^	2.93±1.2^$^	3.12±0.94^$^
I3	Cadaveric dissection as a teaching tool encourages effective teamwork	3.48±0.89	3.40±0.9	3.57±0.72	3.3±0.94	3.45±0.92	3.46±0.88	3.60±0.66
I4	Cadaveric dissection as a learning tool facilitates in the development of communication skills	2.97±1.14	2.91±1.1	3.03±1.05	2.77±1.09	3.08±1.11	2.87±1.19	3.07±0.98
I5	Cadaveric dissection helps the students in developing empathy toward the patient and his problems in later years of medical school	287±1.24	2.66±1.18*	3.1±1.08*	2.61±1.03^$^	2.75±1.21^$^	2.75±1.24^$^	3.13±1.1^$^
TL1	Cadaveric dissection facilitates self-directed learning	3.30±0.86	3.28±0.87	3.34±0.83	3.19±0.88	3.12±0.93	3.33±0.92	3.45±0.75
TL2	Cadaveric dissection facilitates peer-mediated learning	3.30±0.86	3.22±0.86	3.39±0.74	3.35±0.75	3.33±0.71	3.18±1.02	3.33±0.74
TL3	The touch/haptic feedback received during the dissection session provides you with three-dimensional orientations of body structures	3.49±0.87	3.43±0.84	3.55±0.78	3.47±0.67	3.41±0.98	3.4±0.97	3.59±0.66
TL4	Cadaveric dissection facilitates a better understanding of surface and clinical anatomy	3.56±0.85	3.53±0.84	3.59±0.74	3.54±0.8	3.69±0.65	3.53±0.92	3.53±0.79
TL5	The drawing exercise and evaluation at the end of the dissection session enhance the retention of gross anatomy knowledge in medical undergraduate students	3.44±0.92	3.43±0.85	3.47±0.87	3.47±0.74	3.35±0.98	3.44±0.94	3.5±0.79
TL6	The practical assessment methods of anatomy are aligned with the methodology of dissection	2.87±1.26	2.9±1.1	2.84±1.25	2.95±1.02	2.94±1.09	2.82±1.25	2.83±1.24
TL7	The content is too vast during cadaveric dissection sessions for effective memorization	2.18±1.34	2.15±1.25	2.21±1.25	2.28±1.03	2.1±1.32	2.18±1.38	2.17±1.24
DL1	Cadaveric dissection elicits fear and psychological trauma among students	1.17±1.31	1.26±1.24	1.07±1.19	1.16±1.27	1.31±1.35	1.02±1.15	1.18±1.16
DL2	The smell of formalin and the sight of the cadaver creates repulsion from dissection as a learning method	1.71±1.31	1.78±1.17	1.63±1.26	1.63±1.24	1.87±1.25	1.47±1.23	1.8±1.18
DL3	There is a concern regarding health and safety issues while handling cadavers	2.26±1.32	2.4±1.15	2.11±1.31	2.12±1.28	2.43±1.17	2.26±1.27	2.24±1.24
DL4	There is a need to adopt new learning tools for anatomy education such as living anatomy, imaging, and computer-assisted learning instead of cadaveric dissection	2.17±1.51	2.33±1.3	1.97±1.5	2±1.36	2.08±1.37	1.96±1.48	2.41±1.38
DL5	The new teaching approaches, such as living anatomy, imaging, and virtual anatomy, are more cost-effective	2.11±1.46	2.19±1.34	2.03±1.35	2.07±1.1^$^	1.96±1.4^$^	2±1.48^$^	2.28±1.35^$^
DL6	An integrated learning approach by using new learning modes, such as radiological anatomy, living anatomy, and virtual anatomy, along with dissection is more effective than traditional dissection	2.89±1.48	3.11±1.24*	2.64±1.46*	2.44±1.42	2.8±1.47	2.66±1.49	3.28±1.1
DL7	The teaching contact hours allocated to the cadaveric dissection session are very high	1.71±1.41	1.91±1.36	1.48±1.22	1.65±1.17	1.41±1.24	1.75±1.47	1.87±1.3
DL8	The dissection as a learning tool needs to be used only by post-graduate students who are pursuing a career in anatomy and surgical branches	1.24±1.61	1.32±1.55	1.15±1.45	1.19±1.45	1.08±1.41	1.16±1.49	1.4±1.59

Validity and reliability

A reliability analysis of the questionnaire was conducted and Cronbach’s alpha and McDonald's omega values were computed. The mean value of both were 0.865 and 0.884, respectively. Item-reliability statistics were conducted by dropping each item. Cronbach's alpha and McDonald's omega were computed and found to be more than 0.8 for all items (Table 2).

**Table 2:** Reliability index of each item (frequentist individual item reliability statistics) The following items were reverse scaled: TL7, DL1, DL2, DL3, DL4, DL5, DL6, DL7, and DL8. Item codes (questionnaire sections): L1- L4 (learning styles), I1- I5 (interpersonal skills), TL1-TL7 (teaching and learning), and DL1-DL8 (dissection as learning tool)

	If item dropped			
Item	Cronbach's	Item-rest correlation	Mean	SD
L1	0.855	0.642	3.640	0.739
L2	0.853	0.587	2.554	1.235
L3	0.857	0.561	3.612	0.744
L4	0.860	0.403	2.236	1.351
I1	0.855	0.532	2.645	1.211
I2	0.856	0.523	2.884	1.072
I3	0.859	0.431	3.483	0.826
I4	0.857	0.468	2.971	1.076
I5	0.858	0.445	2.868	1.155
TL1	0.856	0.545	3.306	0.853
TL2	0.857	0.533	3.302	0.807
TL3	0.857	0.518	3.488	0.816
TL4	0.857	0.528	3.562	0.793
TL5	0.859	0.451	3.446	0.854
TL6	0.858	0.455	2.872	1.168
TL7	0.870	0.084	1.822	1.248
DL1	0.863	0.290	2.831	1.215
DL2	0.862	0.342	2.293	1.216
DL3	0.863	0.290	1.740	1.233
DL4	0.857	0.487	1.835	1.402
DL5	0.862	0.348	1.888	1.348
DL6	0.865	0.264	1.112	1.366
DL7	0.857	0.476	2.293	1.308
DL8	0.856	0.513	2.760	1.503

Principle components

Principle component analysis identified five components (PC1-PC5) after varimax rotation (Table 3).

**Table 3:** Principal components and their correlations (component loading) The applied rotation method is varimax. Item codes (questionnaire sections): L1-L4 (learning styles), I1-I5 (interpersonal skills), TL1-TL7 (teaching and learning), and DL1-DL8 (dissection as learning tool)

ITEM	PC1	PC2	PC3	PC4	PC5	Uniqueness
L1	0.697					0.342
L2					0.489	0.471
L3	0.690					0.411
L4					0.641	0.462
I1		0.799				0.256
I2		0.833				0.271
I3		0.677				0.410
I4		0.732				0.395
I5		0.505				0.635
TL1	0.515	0.443				0.498
TL2	0.475	0.538				0.476
TL3	0.732					0.406
TL4	0.759					0.362
TL5	0.596				0.518	0.356
TL6					0.612	0.465
TL7					0.413	0.683
DL1				0.757		0.383
DL2				0.775		0.350
DL3				0.752		0.382
DL4			0.743			0.385
DL5			0.728			0.419
DL6			0.657			0.461
DL7			0.649			0.473
DL8			0.562			0.524

**Figure 2:**
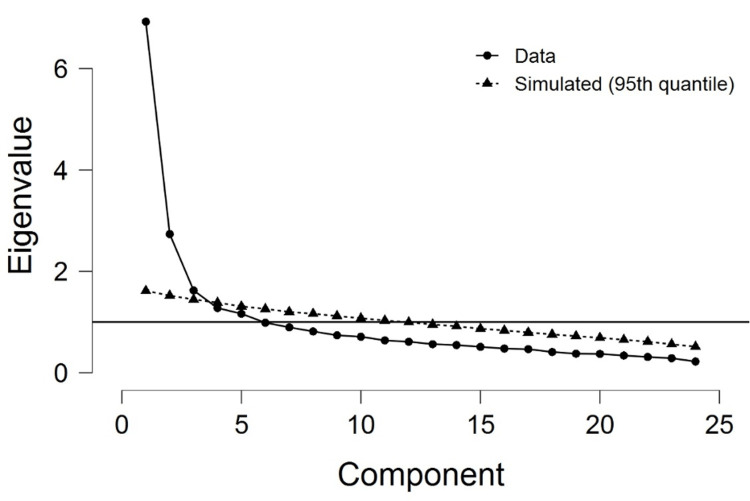
Scree plot and Monte Carlo parallel covariance analysis, based on the magnitude of the associated eigenvalues

The themes, learning ability and structural orientation, interpersonal skills, multimedia and virtual tool, empathy and safety, and associated factors were identified by using principle component analysis from these sections of the questionnaire. Learning ability with structural orientation (PC1: theme 1) and interpersonal skill (PC2: theme 2) have explained 30% of the variance. Multimedia-virtual tool (PC3: theme 3), safety (PC4: theme 4), and associated factors (PC5: theme 5) explained 11%, 9%, and 8%, respectively. Therefore, these five components altogether explained 58% of the variance. The remaining 6-24 items explained 42% of the variance. Based on the magnitude of the associated eigenvalues, the Scree plot, and the Monte Carlo parallel covariance analysis, the first five components are retained and explored (Figure 2).

The examination of the residual covariance matrix did not reveal significant information. The first five components gave a blend of construct variables or items. The mean commonalities were 0.64, which was above the baseline of 0.5. Four themes (PC1, PC2, PC3, and PC5) had a positive relation. They were treated as a latent variable motivation for dissection, and theme 4 (PC4) had a negative correlation and was treated as a latent variable repulsion for dissection (Table 4).

**Table 4:** Principal components or themes PC1-PC5: principal components 1 to 5

Principle components	Construct variable	Latent variable
PC1 (theme 1: learning ability and structural orientation)	Visual learning, kinesthetic learning three-dimensional orientation, surface and clinical anatomy, drawing	Motivation for dissection
PC2 (theme2: interpersonal skill and empathy)	Positive behavior modification, leadership skills, communication skills, teamwork, and peer-mediated learning facilitate in the development of empathy	Motivation for dissection
PC3 (theme 3: multi-media and virtual tool)	Newer learning tools, virtual and imaging anatomy, integrated learning approach, teaching contact hours	Motivation for dissection
PC4 (theme 4: safety and psychological stress)	Formalin repulsion, fear and psychological trauma, safety issues	Repulsion for dissection
PC5 (theme 5: associated factors)	Auditory learning, managing vast content, reading and writing skills	Motivation for dissection

Confirmatory factor analysis

All five themes are examined for their covariances and residual parameters or error terms for estimation shown in Table 5.

**Table 5:** Confirmatory factor analysis (factor covariances)

Confirmatory factor analysis (factor covariances)							
						95% confidence interval	
		Estimate	Std. error	z-value	p	Lower	Upper
Learning ability	↔ Personal skill	0.633	0.050	12.741	<0.001	0.535	0.730
Learning ability	↔ Multimedia-virtual tool	-0.461	0.067	-6.906	<0.001	-0.592	-0.330
Learning ability	↔ Safety	-0.272	0.078	-3.493	<0.001	-0.425	-0.119
Learning ability	↔ Associate factors	0.664	0.063	10.539	<0.001	0.540	0.787
Personal skill	↔ Multimedia-virtual tool	-0.312	0.074	-4.237	<0.001	-0.456	-0.167
Personal skill	↔ Safety	-0.077	0.082	-0.946	0.344	-0.237	0.083
Personal skill	↔ Associate factors	0.640	0.062	10.365	<0.001	0.519	0.761
Multimedia-virtual tool	↔ Safety	0.537	0.071	7.598	<0.001	0.398	0.676
Multimedia-virtual tool	↔Associate factors	-0.539	0.073	-7.34	<0.001	-0.683	-0.395
Safety	↔ Associate factors	-0.253	0.088	-2.862	0.004	-0.426	-0.080
Motivation for dissection	↔ Repulsion for dissection	-0.221	0.077	2.872	0.004	-0.372	-0.070
Motivation for dissection	↔ Multimedia-virtual tool	-0.451	0.065	6.927	<0.001	-0.578	-0.323
Motivation for dissection	↔ Multimedia-virtual tool	0.536	0.071	7.586	<0.001	0.398	0.675

Multimedia-virtual tools and safety negatively correlate with other themes like learning ability, interpersonal skills, and associated factors. Motivational factors (themes 1-3 and 5) also negatively correlate with demotivation factors (theme 4). Demotivational factors have a positive correlation with the use of multimedia-virtual tools.

**Figure 3:**
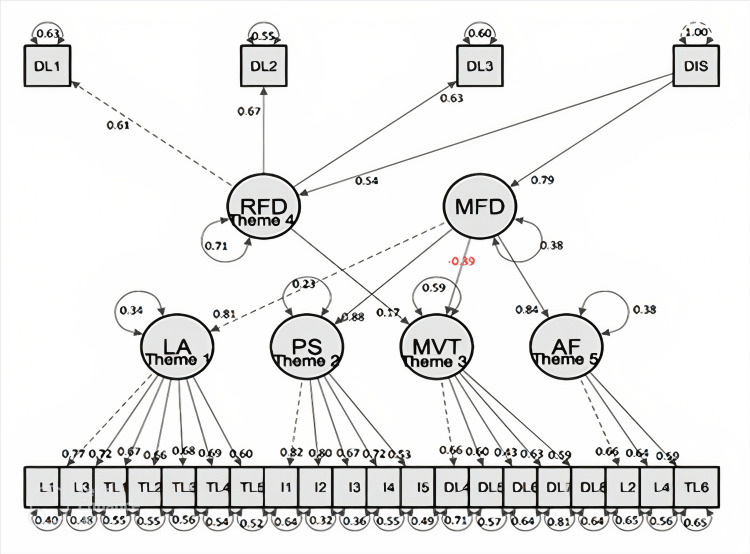
Structural equation model examining the correlation exposure of dissection with motivation and repulsion for dissection. The fit index model: Chi-square comparative fit index (0.922), Tucker-Lewis Index (0.909), standardized root mean square residual (0.061), and root mean square error of approximation (0.54) DL: dissection as learning tool, DIS: dissection, RFD: repulsion for dissection, MFD: motivation for dissection, LA: learning ability, PS: personal skills, MVT: multimedia-virtual tool, AF: associate factors, L1-TL6: questionnaire item numbers

The structural equation model (Figure 3) revealed that the exposure to cadaveric dissection (duration) strongly correlated (0.79) with motivation for dissection, but it also moderately correlated (0.54) with repulsion for dissection due to safety in some individuals (theme 4). Therefore, exposure to cadaveric dissection caused significant repulsion due to formalin smell and safety factors. Learning ability, structural orientation (theme 1), and interpersonal skills (theme 2) explained more than 30% of the variance of learning with dissection. They are correlated with motivation (MFD), and the correlation coefficients were 0.81 and 0.88, respectively. The exposure to dissection promoted learning ability and structure orientation along with interpersonal skills. Though multimedia and virtual tools promoted the use of cadaveric dissection, an acquaintance of cadaveric dissection had a negative correlation (-0.39) with further use of multimedia or virtual tools. Associated factors (theme 5), like auditory learning, reading and writing skills, and managing vast content, also motivate cadaveric dissection.

## Discussion

Anatomy is primarily taught in India using dissection and prosection. The favored technique of teaching anatomy by the concerned faculty is still cadaveric dissection, despite the availability of more modern, advanced technologies [19]. Acknowledging the imperative merit and applicability of dissection in anatomy teaching and learning, this study's main aim is to examine the role of cadaveric dissection in teaching the subject based on faculties perceptions.

Gender variation

Descriptive statistics exhibited the least gender variation in most of the survey items, except empathy and positive behavior. A study by Akpan and Andre found no gender difference in the impact of dissection [20]. Another study examined student perceptions and indicated that male students had a higher motivation than females in cadaveric dissection experience [21]. Similar findings were also reported by Hancock et al. who documented that females had the most frequent anxiety, stress, and other psychological issues [22]. The females react more sensitively than males in the dissection room [23]. The dissection hall visit, discussion about cadaveric donation and dissection, and cadaveric dissection videos were useful strategies to address these issues [24]. A student’s advisor would also help in reducing fear and anxiety [25]. Medical education should include helping female students to deal with their increased susceptibility to stress during dissection sessions [26].

Theme 1

In theme 1, learning ability and structural orientation include visual learning, kinesthetic learning, three-dimensional orientation, surface and clinical anatomy, and drawing exercises done in the dissection session. Visual, auditory, read/write, and kinesthetic sensory modalities are represented by the characters VARK. When presenting information, figures and infographics are favored by visual learners. For auditory learners, lectures and discussions are the best ways to hear information. Readers prefer text-based content. Kinesthetic learners prefer practical exercises that relate the subject to context-based hands-on learning. Research studies showed that cadaveric dissection mapped with VARK learning styles as a teaching and learning method fosters both visual and kinesthetic learners [27]. Studies also showed that learning styles influence both the study span and scores of students in anatomy courses [28]. Therefore, the incorporation of drawing exercises in dissection sessions has a positive effect in modifying students' learning styles and facilitating achieving the learning outcomes [28-29]. Student perceptions in a study done by Jeyakumar et al. (2020) showed that cadaveric dissection influenced the knowledge of applied anatomy and facilitated the development of clinical skills [30]. The results of this study showed a positive correlation between the motivation for dissection with learning ability and structural orientation [31]. Research underlined that they were able to better comprehend how things "fit together" and had a respect for the three-dimensional image of the anatomy of the body [31]. They also reiterated cadaveric dissection's notable role in three-dimensional learning anatomy, kinesthetic-mediated skills, and anatomical variations. Similar perceptions from the students are also there to support the above viewpoints [14-18].

Theme 2

Theme 2 (interpersonal skills and empathy) identified in the principal component analysis included positive behavior modification, leadership skills, communication skills, teamwork, peer-mediated learning, and facilitating the development of empathy from the construct variables. The development of communications skills, teamwork, and facilitating peer learning has been one of the pedagogical advantages of cadaveric dissection done in small groups [31]. Previous studies exhibited that the activity of peer-mediated learning not only allowed students to share their experiences and learning with each other but can also improve assessment performance [32-33]. This contention is supported by current findings, at least in part. The dissection room provides a cooperative learning environment for positive behavior modification, communication skills, and leadership skills in such a team-based or case-based approach. Such personality modification in learners showed gains in student performance when used effectively [34]. The beneficial effects on personality growth among the learners have been found while participating in dissection [31]. It may be difficult to figure out, however, whether their responses are limited to the effects of dissection and working in that environment or are rooted in a wider understanding of what it means to be a part of the medical school community. Students had the ability to consider their futures and prospective areas of specialization after being exposed to dissection. The dissection is advantageous for students who are selecting their future professions [35]. The motivation score and the degree of anatomical knowledge were found to be positively correlated. Competence or mastery is one of the crucial components that maximize intrinsic motivation, as mentioned in the literature [36-37]. To encourage intrinsic motivation, it is important to satisfy students' psychological requirements for autonomy, competence, and relatedness [36]. For higher performance, students with internal motivation were found to have greater interest, confidence, and tenacity as well as more deep-level learning strategies [38]. Positive feedback enhances intrinsic motivation and performance more than negative input [39]. Personality-type psychological profiling may be useful in this approach [40].

Theme 3

Theme 3 (multi-media and virtual tools) include newer learning tools, virtual and imaging anatomy, and integrated learning approaches (Figure 1). Studies showed that multimedia and virtual tools are effective learning tools as a supplement with cadaveric dissection that facilitate understanding complex anatomical structures with effective motivational levels among learners [41-42]. The multimedia or virtual tools have motivated learners to initiate cadaveric dissection, but a weak significant negative correlation was observed on further use of multimedia tools or simulation technologies. Most of the studies reported the positive effects of simulation on anatomy learning as measured by digital images of anatomical structures [43]. This caveat is important as the findings of the present study are in contradiction with others. There could be two possibilities: the first one is inadequate familiarity with the multimedia tools, simulation technologies, and the ability to transfer the knowledge learned with a multimedia simulation technology to actual human beings. There is controversy in anatomy instruction about the extent of knowledge students can apply to a real scenario. Nasr (2007) and Hisley et al. (2008) suggested that they could not transfer their knowledge of anatomical structures in a real scenario [43-44]. Students learning with multimedia tools were disadvantaged because they were unable to transfer their knowledge to actual human cadavers [45]. The results of the current study underscore the need for more investigation into how and when to combine multimedia simulation experiences with genuine cadaver-based experiences, as many anatomy educators may and will use modern technology to enhance traditional laboratories [46].

Theme 4

Theme 4 (safety and psychological stress) identified the negative questions from the questionnaire in principal component analysis that includes questions on formalin repulsion, safety issues, and fear and psychological trauma induced due to exposure to the cadaver. The initial dissection room stresses before the commencement of dissection. However, most medical students perceive cadaver dissection to be a significant and positive life event [47]. Studies showed that medical students during early encounters experience fear, psychological stress, and eye irritation as well as repulsion from the smell of formaldehyde [48]. It has been evident that anxiety is the common component on first exposure of the students to the cadaver; however, repeated exposures and pre-session induction by showing dissection videos and pictures facilitate in allaying the element of anxiety [49-50]. Due to the cost and effectiveness, formalin remained the main fixative for cadavers used in Indian medical schools; however, it has been recommended to create awareness among staff members and students about the usage and preventive measure [51]. In this study, similar findings have been observed that the repulsion from dissection is due to formalin and safety factors.

Theme 5

Theme 5 (associated factors) identified the questions related to auditory learning, management of vast content, reading, and writing skills. These factors were found to be positively correlated with motivation for dissection. Auditory learning, reading, and writing skills are not directly addressed during the dissection process, but the discussions generated during the session among peers and tutors on the dissection table facilitate auditory learning [27].

Educational implications

The findings of this study provide useful guidance for selecting and developing motivational methods and strategies. A crucial stage in determining the motivational elements and the features of the learners is doing a perception study of the faculties. The latter include gender distinctions, historical context, and dissection exposure. Learning about the various motivational variables that are represented by the five themes of this approach is essential. The creation of motivating goals and the decision on the best techniques to reach those goals are guided by this knowledge. For instance, several motivational tactics, such as active engagement, variability, conflict, and inquiry based on the attention, relevance, confidence, and satisfaction model, may be employed to increase motivation [52]. If a student's overall motivation is strong, the teacher can maintain this level of motivation by employing multiple teaching modalities and giving the right kinds of motivational feedback. The aforementioned elements provide a learning environment that resonates with the requirements of the students [53-54].

## Conclusions

The dissection room is an important place for learning clinical and personal skills along with empathy. There has never been any uncertainty about the relevance of motivation in learning. The current model provides validated information about motivational factors for cadaveric dissection. The cadaveric dissection experience and associated learning activities significantly impact students’ motivation, which confirms the important value of teaching anatomy using cadavers. These results are important for selecting appropriate strategies for motivational design. This study also concluded that cadaveric dissection holds its place as an effective learning tool for anatomy education in the Indian context. The studies showed that instructional approaches must be integrated in a manner that will foster all the learning styles of VARK. Using mixed-method approaches (Figure 1) that integrate new approaches to learning anatomy (virtual anatomy, living anatomy, and radiological anatomy), along with cadaveric dissection. will be a way forward in this context. Instructional methods, such as problem-based learning sessions, need to be incorporated where anatomy will be learned with interdisciplinary integration, and an integrated resource center that includes resources from a human cadaver to the virtual tools will provide multimodal stimulation to the learners by addressing diverse learning styles. Furthermore, there is a need to address the safety issues and implementation of stress-coping activities during the induction phase. It will also foster building and achieving broader undergraduate Indian medical curricula competencies such as leadership, communication, and teamwork from the early days. The primary limitations of this study include its lack of comparison to alternative laboratory teaching techniques (such as virtual dissection or prosection) and its failure to establish a connection between students' motivational scores and performance information (course and laboratory examination scores). The fact that multiple tasks are performed during the dissection in the lab may be another study restriction because it will be challenging to relate the results solely to the dissection itself.
